# Structural basis for plazomicin antibiotic action and resistance

**DOI:** 10.1038/s42003-021-02261-4

**Published:** 2021-06-11

**Authors:** Tolou Golkar, Angelia V. Bassenden, Krishnagopal Maiti, Dev P. Arya, T. Martin Schmeing, Albert M. Berghuis

**Affiliations:** 1 Department of Biochemistry, McGill University, McIntyre Medical Building, Montréal, QC Canada; 2 Centre de Recherche en Biologie Structurale, McGill University, Bellini Life Science Complex, Montréal, QC Canada; 3 Department of Chemistry, Clemson University, Clemson, SC USA; 4 Department of Microbiology and Immunology, McGill University, Duff Medical Building, Montréal, QC Canada

**Keywords:** Ribosomal proteins, X-ray crystallography, Transferases

## Abstract

The approval of plazomicin broadened the clinical library of aminoglycosides available for use against emerging bacterial pathogens. Contrarily to other aminoglycosides, resistance to plazomicin is limited; still, instances of resistance have been reported in clinical settings. Here, we present structural insights into the mechanism of plazomicin action and the mechanisms of clinical resistance. The structural data reveal that plazomicin exclusively binds to the 16S ribosomal A site, where it likely interferes with the fidelity of mRNA translation. The unique extensions to the core aminoglycoside scaffold incorporated into the structure of plazomicin do not interfere with ribosome binding, which is analogously seen in the binding of this antibiotic to the AAC(2′)-Ia resistance enzyme. The data provides a structural rationale for resistance conferred by drug acetylation and ribosome methylation, i.e., the two mechanisms of resistance observed clinically. Finally, the crystal structures of plazomicin in complex with both its target and the clinically relevant resistance factor provide a roadmap for next-generation drug development that aims to ameliorate the impact of antibiotic resistance.

## Abstract (web summary)

Golkar, Bassenden et al. report two structures of the latest generation aminoglycoside antibiotic plazomicin in complex with the bacterial 70S ribosome as well as in complex with AAC(2’)-la acetyltransferase, an antibiotic modification enzyme (AME). Their study can be useful in the development of newer aminoglycosides that are not modified by AMEs while being capable of targeting the bacterial ribosome.

## Introduction

It is widely recognized that antibiotic resistance poses a serious threat to global public health. The high consumption of antibiotics in our food chain and health care systems has drastically waned the effectiveness of antibacterial treatments, severely compromising our ability to manage infections^1^. Despite numerous programs to reduce usage and control prescription, resistance to clinically used antibiotics remains widespread, and the number of bacterial pathogens presenting multidrug resistance continues to rise^1^.

To alleviate the pressure on our current armament of antibiotics, much effort has been directed at creating new treatment options^2^. The results from these efforts have thus far been limited, highlighting the difficulties in developing new antibiotics in the context of resistance^3^. However, a newly developed aminoglycoside antibiotic, plazomicin (marketed as Zemdri), was approved for clinical use by the U.S. Food and Drug Administration (FDA) in 2018; and since then, sister agencies in other countries have also approved its use. Like other aminoglycosides, plazomicin binds to the 16S rRNA at the aminoacyl-tRNA site (A-site) of the 30S ribosomal subunit, interfering with protein translation^4,5^. Plazomicin’s in vitro activity displays similar MIC ranges against Gram-negative and Gram-positive bacteria as other commonly used aminoglycosides, such as gentamicin, tobramycin, and amikacin^6–13^. Clinical studies have proven plazomicin effective in the treatment of complicated urinary tract infections and pyelonephritis^14^ and have shown activity against emerging clinical drug-resistant bacteria, including *Enterobacteriaceae*, *Pseudomonas aeruginosa*, and *Staphylococcus* spp, such as methicillin-resistance *Staphylococcus aureus*^7,12^.

Chemically, plazomicin is derived from sisomicin, an aminoglycoside that closely resembles gentamicin, with synthetic modifications incorporated at the N1 and N6′ positions of the antibiotic^15^. The N1 position is extended by appending a hydroxy-aminobutyric acid (HABA) substituent, and the N6′ is modified through the addition of a hydroxyethyl (HE) substituent. The presence of these chemical alterations allows plazomicin to evade the action of nearly all clinically relevant resistance mechanisms, which are largely mediated by aminoglycoside modifying enzymes (AMEs)^13^. Notably, plazomicin is impervious to the action of AAC(3) and AAC(6′), the most common aminoglycoside acetyltransferases in *P. aeruginosa*^16^, as well as ANT(2′′) and APH(2′′), the most common AMEs in the *Enterobacteriaceae* family^17^. Plazomicin also lacks hydroxyl groups at the 3′ and 4′ positions, protecting it against the activity of AMEs ANT(4′) and APH(3′)^6^. Although the chemical modifications incorporated in the structure of plazomicin substantially increase its resilience against the activities of most AMEs, this antibiotic has shown to be still susceptible to the action of enzymes capable of modifying amino moieties at the 2′ position. Specifically, AAC(2′)-Ia is reported to cause plazomicin resistance at elevated minimum inhibitory concentrations (MIC)^9^. In addition, plazomicin is incapable of circumventing some of the target alteration mechanisms of resistance, mediated by 16S ribosomal methyltransferases^8–11^.

Here, we present the crystal structure of plazomicin bound to its target the 70S ribosome in complex with mRNA and tRNAs. This structure sheds light on the structural basis for plazomicin’s antibiotic properties and provides insights into the effectiveness of target alteration-based resistance mechanisms. Additionally, the crystal structure of inactivated plazomicin in complex with AAC(2′)-Ia is presented. This structural information combined with that from the plazomicin bound ribosome provides foundational data for addressing resistance to one of the newest antibiotics presently available for clinical use.

## Results

### Structure of plazomicin bound to the Ribosome–mRNA–tRNAs complex

The crystal structure of the *Thermus thermophilus* ribosome in complex with plazomicin was determined to 3.27 Å. The crystal form used for this was previously exploited for the elucidation of the interactions between the ribosome and several other antibiotics^18,19^ and contains two copies of the 70S ribosomes in complex with mRNA and three tRNAs in the asymmetric unit. As has been observed for the other isomorphous crystal structures^18,19^, the presented 70S ribosome complex structure does not contain two of the ribosomal proteins bL12 and bS1. Also, a disorder in some of the components is noted; most relevant, the tRNA positioned in the E-site contains segments that could not be modeled due to disorder. Data collection details and final refinement statistics are given in Table 1.

**Table 1:** Data collection and refinement statistics.

	*T. thermophilus* ribosome–Plazomicin	AAC(2′)-Ia–CoA–Acetylated Plazomicin
*Data collection*		
Space group	P 21 21 21	P 32 2 1
*Cell dimensions*		
*a*, *b*, *c* (Å)	209.5, 449.4, 619.6	73.5, 73.5 147.1
*α*, *β*, *γ* (°)	90, 90, 90	90, 90, 120
Resolution (Å)	127.5–3.27 (3.38–3.27)^a^	58.43–1.95 (2.02–1.95)
*R*_merge_	0.214 (1.27)	0.052 (0.96)
*I*/σ*I*	5.94 (1.2)	18.9 (2.1)
Completeness (%)	99.7 (97.5)	98.8 (97.5)
Redundancy	6.5 (5.4)	8.1 (7.0)
*Refinement*		
Total no. of reflections	5,816,392 (463,077)	273,465 (23,176)
*R*_work_/*R*_free_	0.214/0.277	0.192/0.225
No. of atoms	296,449	3096
Macromolecules	294,983	2756
Ligand/ion	1464	224
Water	2	116
*B*-factors	86.8	48.9
Macromolecules	87.0	46.9
Ligand/ion	61.7	58.0
Water	56.9	47.4
*R.m.s. deviations*		
Bond lengths (Å)	0.012	0.006
Bond angles (°)	1.82	0.80

One crystal used for data collection of each structure.

^a^Values in parentheses are for the highest-resolution shell.

Examination of discovery maps for the ribosome complex unambiguously identified that plazomicin binds to the highly conserved decoding region of the aminoacyl-tRNA site (A-site) on 16S rRNA in both ribosome complexes in the asymmetric unit (Fig. S1a). Specifically, plazomicin binds in the major groove of the 16S rRNA of the small ribosomal subunit at the base of helix 44, where two conserved adenine residues at positions 1492 and 1493 (*Escherichia coli* numbering) flip out of the helix (Fig. S2 and Fig. S3). This site corresponds to what had been predicted based on resistance conferred by ribosomal methyltransferases and also corresponds to where structurally related aminoglycosides interact with the bacterial ribosome^20^. It is noteworthy that in some crystal structures of aminoglycoside ribosome complexes, a second aminoglycoside binding site has been identified, i.e., helix 69 of the 23S rRNA of the large ribosomal subunit^21^. However, the structure presented here does not reveal any additional binding sites for plazomicin beyond the ribosomal A-site.

**Fig. 1:**
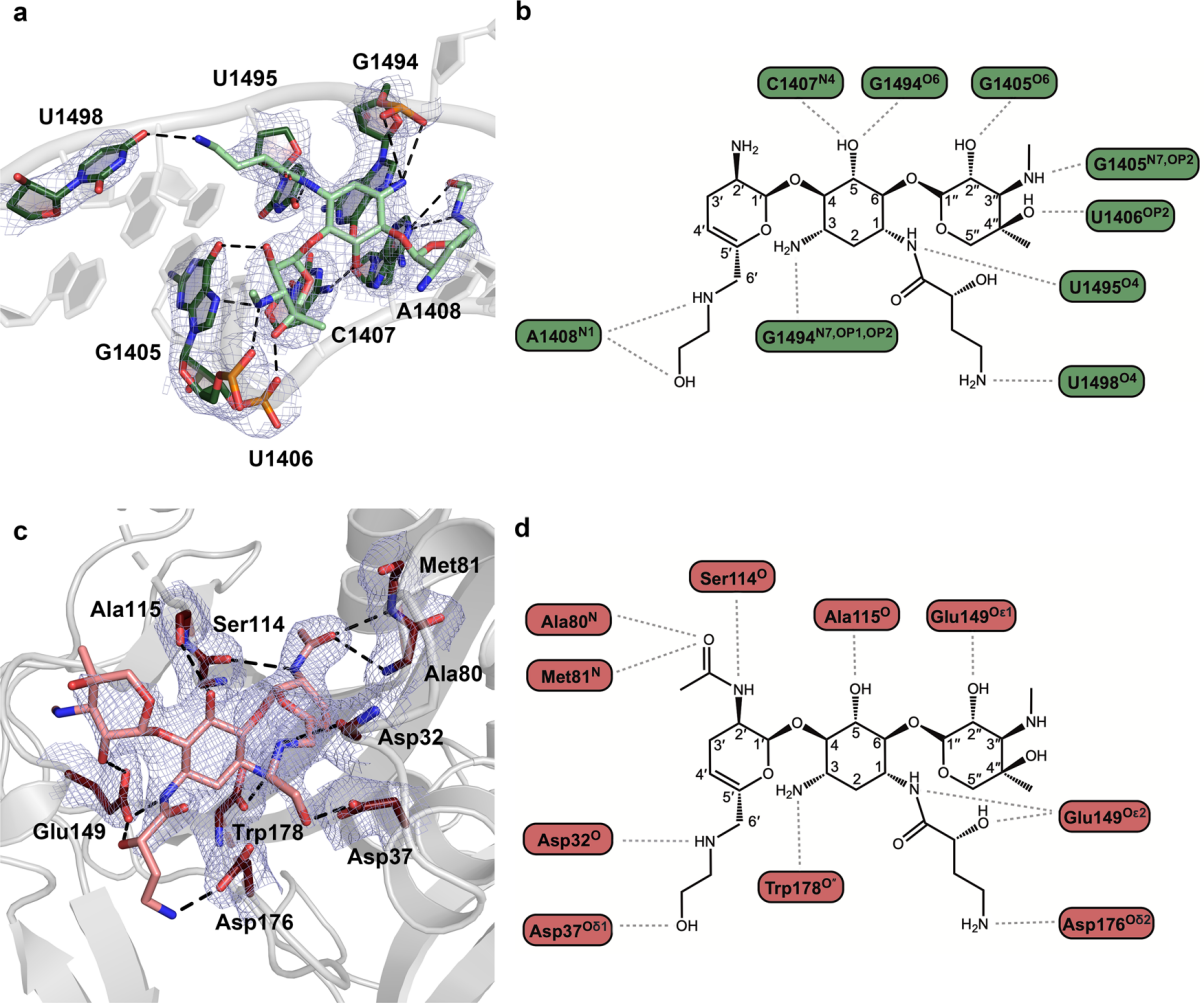
The ribosomal A-site and AAC(2′)-Ia hydrogen bond interactions with plazomicin. **a** Ribosomal A-site bases involved in interactions depicted as sticks and colored in dark green, plazomicin colored in light green. Hydrogen bonds are depicted as black dashed lines. The composite 2*F*_o_–*F*_c_ map is contoured to 1*σ* and colored in blue. **b** A 2-dimensional representation of hydrogen bond interactions between plazomicin and the ribosomal A-site. **c** AAC(2′)-Ia residues involved in interactions depicted as sticks and colored in dark red, acetylated plazomicin colored in salmon. The composite 2*F*_o_–*F*_c_ map is depicted as in (**a**). **d** A 2-dimensional representation of hydrogen bond interactions between acetylated-plazomicin and AAC(2′)-Ia.

The crystal structure allowed for the identification of specific interactions between plazomicin and the rRNA (Fig. 1a, b). The N1 and N3 amino groups on the central ring of the aminoglycoside interact with nucleotides G1494 and U1495, respectively, while the O5 hydroxyl on the central ring interacts with nucleotides C1407 and G1494. Furthermore, the synthetically added HABA tail of plazomicin forms a hydrogen bond with the uracil base of U1498. Moreover, the stacked arrangement of plazomicin’s prime ring and the purine ring of G1491 allows the hydroxyl and amino groups on the 6′-HE tail to form a pseudo-base-pair with A1408. Finally, the double-prime ring forms hydrogen bonds to the Hoogsteen sites (N7 and O6) of nucleotide G1405, as well as phosphate oxygens of U1405 and U1406.

A comparison of plazomicin binding to the ribosomal A-site with other related 4,6-disubstituted aminoglycosides, including gentamicin, tobramycin, and amikacin shows that the binding mode of these aminoglycosides shares many similarities. Most notably, the interactions made by the central deoxystreptamine ring are highly conserved. Moreover, plazomicin and amikacin show similar conformation in their shared HABA synthetic additions at their N1 positions. However, the conformation of the prime ring in plazomicin is slightly different from the other ribosome-bound aminoglycosides due to the contribution of the hydroxyl group on the HE tail of plazomicin in pseudo-base-pair formation between the prime ring and A1408 (Fig. S4).

### Structure of inactivated plazomicin bound to AAC(2′)-Ia

The high-resolution crystal structure of acetylated-plazomicin and CoA bound to AAC(2′)-Ia from *Providencia stuartii* was determined at 2.0 Å. The overall structure of the enzyme has been previously reported in a different crystal form with different ligands^13^. Also, we have reported structures of AAC(2′)-Ia in complex with different aminoglycosides that employ the same crystal form used here^22^. As expected, there are no major differences observed in the fold of the enzyme in all of these various AAC(2′)-Ia structures. Also, the structures all confirm AAC(2′)-Ia exists as a homodimer under physiological conditions, as is anticipated for the AAC class of AMEs^23^. The data collection details and final refinement statistics for this crystal structure are given in Table 1.

Crystals of AAC(2′)-Ia were grown in the presence of the substrates plazomicin and acetyl-CoA. However, discovery maps unequivocally identified the enzymatically modified plazomicin and CoA in the active site in each unit of the dimeric structure, indicating that the acetylation reaction had occurred during crystallization and that the product bound state of the enzyme was captured (Fig. S1b, S1c).

The AAC(2′)-Ia plazomicin binding pocket primarily wraps around the central and prime rings of the aminoglycoside, while the double-prime ring is relatively solvent-exposed. The pattern of hydrogen bonds between AAC(2′)-Ia and acetylated-plazomicin reveals that the majority of interactions occur at the central ring and the prime ring, the latter being the site of 2′-aminoglycoside modification (Fig. 1c, d). Although the enzyme forms few interactions with the double-prime ring of plazomicin, Glu149 forms an interaction with the 2′′-hydroxyl group of the aminoglycoside. At the central ring, the enzyme forms hydrogen-bond interactions with the 3-amine and 5-hydroxyl groups of plazomicin using Trp178 and Ala115, respectively. At the prime ring, the majority of the interactions take place at the 2′-site of modification. The Ser114 interacts with the 2′-amine, while residues Ala80 and Met81 interact with the oxygen of the 2′-acetyl modification. Of particular interest is AAC(2′)-Ia’s ability to accommodate the two synthetic additions of plazomicin, as it is this feature that allows the enzyme to confer resistance to the newest aminoglycoside antibiotic. The N1 HABA tail extends away from the central ring in a solvent-exposed region, though the N1 secondary amine moiety forms hydrogen bonds with Glu149 and Asp176. The N6′ HE extension sits in a crescent-shaped tunnel of the enzyme and forms a hydrogen bond with residues Asp32 and 37.

### Comparison of plazomicin binding to target vs. resistance factor

**Fig. 2:**
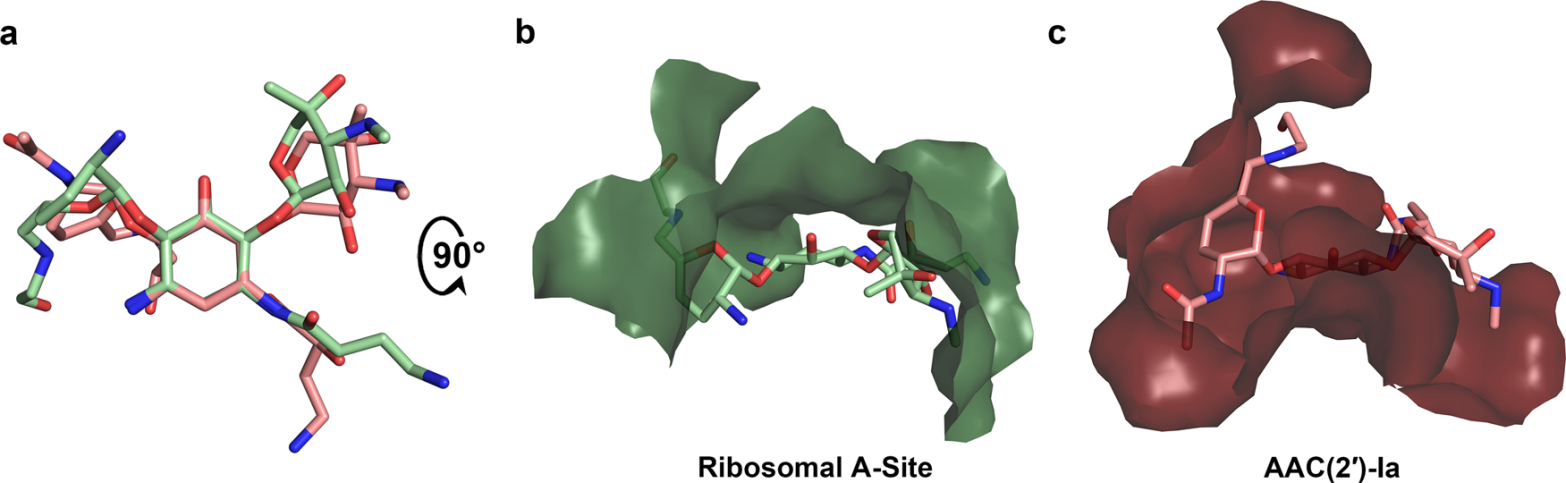
Comparison of plazomicin and acetylated-plazomicin binding to the ribosomal A-site and AAC(2′)-Ia. **a** Overlay of ribosome-bound plazomicin (light green) and the AAC(2′)-Ia-bound acetylated-plazomicin (salmon) using the aminoglycoside’s central ring as the common structural motif. **b** Plazomicin binding site in the ribosomal A-site. **c** Acetylated-plazomicin binding site in AAC(2′)-Ia. Perspective is flipped 90˚ from panel (**a**) in panels (**b**) and (**c**). The color scheme is as per Fig. 1.

There is much similarity observed in how plazomicin interacts with the ribosome in comparison with its clinically relevant resistance enzyme AAC(2′)-Ia. First, the conformation of plazomicin and the inactivated acetylated plazomicin is very similar, with the main differences being rotations of ~15–35° in the four glycosidic bonds that connect the prime ring and double-prime ring to the central deoxystreptamine ring, culminating in ~60° and ~40° hinge rotations for the prime and double-prime rings, respectively. In addition, a 180° flip in how the HABA tail links to the N1 group is noted (Fig. 2a). Secondly, nearly all of the hydrogen bonds formed by the latest aminoglycoside with its target are conserved in the structure of the AAC(2′)-Ia resistance factor (Fig. 1). The similarities in both aminoglycoside conformation and hydrogen bond interactions in the ribosome and various AMEs have previously been noted for naturally occurring aminoglycosides, such as kanamycin and gentamicin^24,25^. While there are striking similarities in the binding pose and hydrogen bond interactions, the van der Waals interactions made by plazomicin with the 16S rRNA bears little resemblance to how this same antibiotic interacts with AAC(2′)-Ia. In fact, most of the van der Waals interactions made by these two macromolecules are at opposite faces of the antibiotic (Fig. 2b, c). Substantial differences in van der Waals interactions have also been seen when examining aminoglycoside interactions with several other AMEs^24,25^. Importantly, it is the substantial differences in van der Waals interactions among AMEs, specifically with respect to the ribosome, that enables plazomicin to evade resistance by, for example, AAC(3), ANT(2′′), and APH(2′′)^6^.

## Discussion

The determination of the three-dimensional structures of both the ribosome complex and AAC(2′)-Ia bound to the newest aminoglycoside antibiotic to near-atomic resolution allows for mechanistic analysis of how plazomicin exerts a bactericidal effect and how clinically relevant resistance is achieved. In turn, this information can be exploited for the design of next-generation aminoglycosides that are less susceptible to existing methods of resistance.

The structure of plazomicin bound to the 70S bacterial ribosome in complex with mRNA and tRNAs reveals that it specifically binds to the 16S rRNA ribosomal A-site (Fig. S2). This site also coincides with the physiologically relevant binding site for plazomicin, confirmed by resistance-conferring ribosomal methylation sites, which all concentrate at this location (see below). The structural consequence of plazomicin binding is that bases A1492 and A1493 are extruded from helix 44 of the 16S rRNA. This conformation of the ribosomal A-site resembles the state in which the codon-anticodon helix is recognized through a minor groove interaction and enables cognate tRNA accommodation^26,27^. Locking the ribosomal A-site in this conformational state following plazomicin binding can, therefore, induce the incorporation of near- and non-cognate tRNAs into the ribosome during the decoding process^4^. The overall impact is that the fidelity of mRNA translation is compromised through the binding of plazomicin. It has been speculated that the resultant production of aberrant proteins induces stress on bacteria, including compromised membrane integrity, which ultimately precipitates a bactericidal effect^28^. This mode of action is identical to what has been proposed for other aminoglycosides that bind to the ribosomal A-site^4^.

An additional mechanism by which aminoglycosides exert antibiotic activity has been proposed, i.e., inhibition of ribosome recycling via binding to helix 69 of the 23S rRNA^21^. As mentioned above, the structure of the plazomicin ribosome complex does not reveal aminoglycoside binding in helix 69. Moreover, modeling of plazomicin into this location based on the gentamicin binding pose reveals this to be impossible due to predicted steric clashes of the N1 HABA extension with G1910, U1911, C1920, and G1921 (Fig. S5). Therefore, based on structural data, it is unlikely that plazomicin interferes with ribosome recycling.

Resistance to plazomicin has been noted through two main mechanisms: drug modification and target alteration. The clinically identified mechanism of drug modification is the acetylation of plazomicin at the 2′ position catalyzed by AAC(2′)-Ia^15^. Clinically, observed plazomicin resistance through target alteration has been affected by ribosomal 16S rRNA methylation, specifically methylation of G1405 by enzymes such as ArmA^8^.

While there are well over 100 different AMEs that have been identified in pathogenic bacteria, making covalent modification of aminoglycosides the most prominent mechanism of resistance to this class of antibiotics, AAC(2′)-Ia is unique in that it is presently the only AME that can efficiently use plazomicin as a substrate^29^. The structure of the plazomicin enzyme complex shows the reason for this, i.e., the aminoglycoside binding pocket of AAC(2′)-Ia can accommodate both the HABA and HE extensions, while the enzyme remains perfectly poised to modify one of the functional groups on the antibiotic. Other AMEs may be able to accommodate one or both of the synthetic extensions of plazomicin, but this is invariably accompanied by a dramatic reduction in enzyme efficiency. For example, APH(2′′)-Ia has been shown to accept aminoglycosides containing the HABA tail, but this coincides with a compromised ability to phosphorylate these antibiotics^30^.

**Fig. 3:**
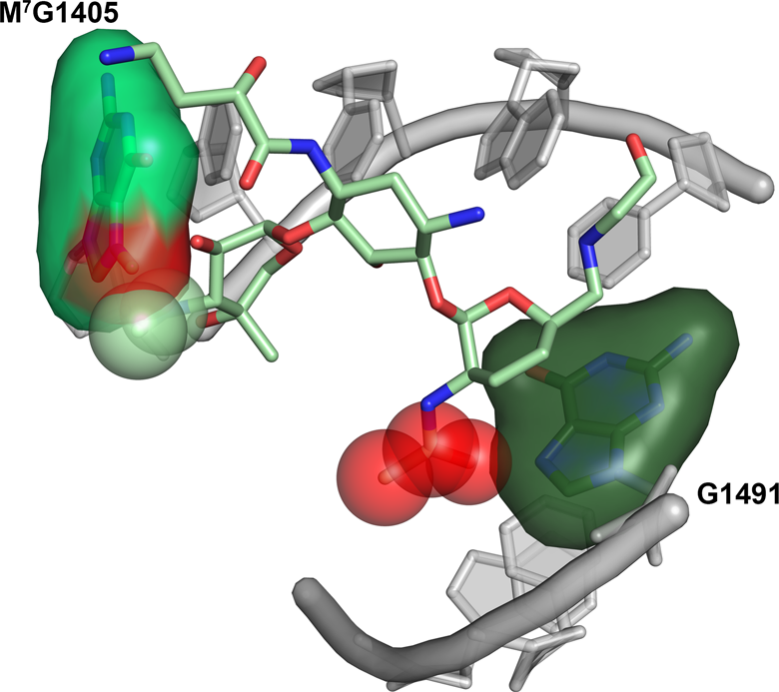
Ribosome methylation and acetylated plazomicin clashes. Methylation and acetylation sites are colored in red. M^7^G1405 is shown as a surface colored in neon green clashing with the 3′′ group of plazomicin, shown as spheres. G1491 is shown as a surface colored in dark green clashing with the acetyl group of plazomicin, shown as spheres.

The structure of the plazomicin bound ribosome complex sheds light on the consequences of 2′ acetylation for the antibiotic properties of this aminoglycoside. Modeling of the inactivated plazomicin into the ribosomal A-site reveals that the carbonyl group of the additional acetyl moiety would inevitably cause steric clashes with O6 and/or N7 of G1491 (Fig. 3). It is conceivable that the extent of the steric clash can be reduced by allowing for substantial conformational strain in the acetylated plazomicin structure, but the overall energetics would remain unfavorable. Moreover, this steric clash is aggravated by the actuality that all the groups involved in interactions are hydrogen acceptors, including G1491 N7, implying that the loss of water-mediated hydrogen bonds upon 2′-acetylated-plazomicin binding cannot be compensated by new hydrogen bonds between the acyl carbonyl group and G1491. Finally, the 2′ amino group in plazomicin is most likely protonated, creating a positive charge at this site that forms favorable charge interactions with three negatively charged phosphate backbone groups that are positioned within 7 Å. Upon acetylation, the charge on the 2′ group is removed, abolishing this favorable charge interaction. While separately the steric clash/strain, loss of hydrogen bonds, and loss of charge interactions may be insufficient to prevent binding of acetylated plazomicin; together, these three factors result in 2′-acetylation by AAC(2′)-Ia to confer resistance to plazomicin.

Various 16S rRNA methyltransferases mediate the methylation of the N7 position of G1405 resulting in m^7^G1405, which precipitates resistance to plazomicin. Most notable is ArmA, which is found in *Enterobacteriaceae* family including *Klebsiella pneumoniae*^10,11^. The plazomicin bound ribosome complex structure, again, enables the rationalization of why the addition of a methyl group to a select RNA base confers resistance. Modeling of m^7^G1405 reveals that, in addition to abolishing the potential hydrogen bond between N7 and the secondary amine on the plazomicin double-prime ring, the methylation would also create severe steric clashes with this ring (Fig. 3). An additional aspect of methylation is that this modification introduces a positive charge within the ribosomal A-site, which is generally unfavorable for promoting interactions with aminoglycosides, given their predominantly positive charge. This charge contribution to effecting resistance echoes that of acetylation, where a positive charge on the antibiotic is removed. The modeling of the impact of the m^7^G1405 alteration on resistance for other aminoglycosides that target the ribosomal A-site mirrors the explanation provided here^31^.

Much of next-generation aminoglycoside development has exploited two complementary strategies: the removal of functional groups so as to circumvent modification by AMEs, and the addition of synthetic extensions so as to interfere with AME binding. However, both strategies have caveats since many of the functional groups are required for ribosomal A-site binding, and extensions on the core chemical structure can also prevent binding to the 16S rRNA. The development of plazomicin successfully used both strategies by using sisomicin as its core, lacking functional groups on the 3′ and 4′ positions and incorporating extensions on the N1 and N6′ positions. Nonetheless, both 2′ acetylation of plazomicin and G1405 methylation cause high-level resistance.

**Fig. 4:**
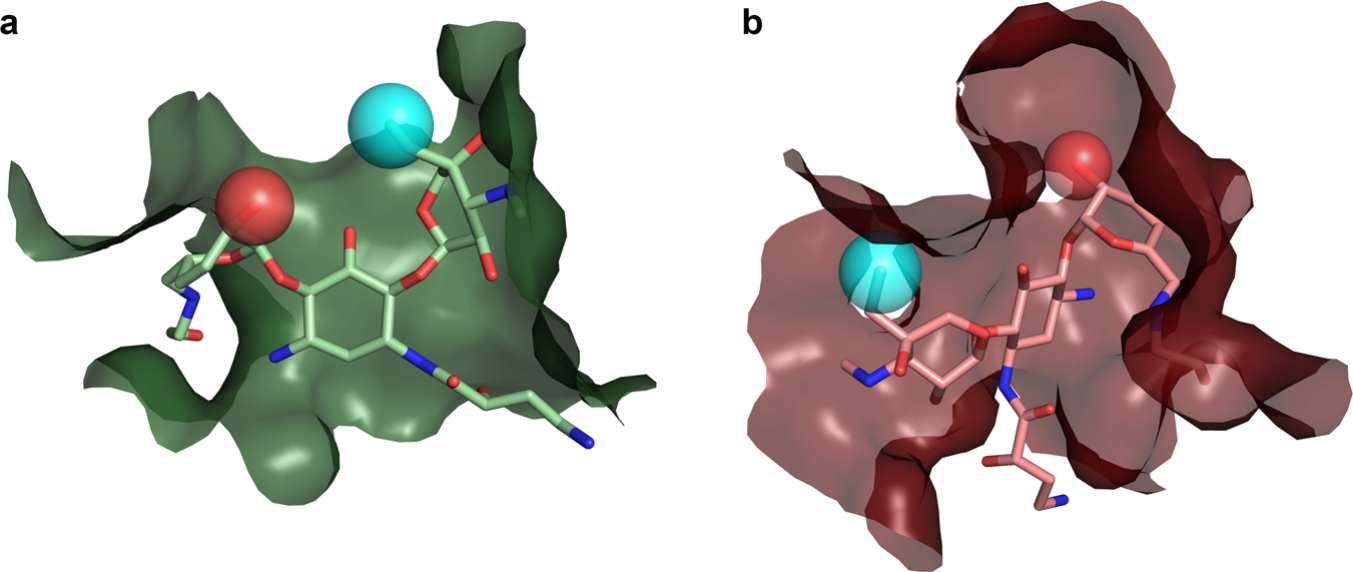
Proposed sites for next-generation aminoglycoside synthesis. Shown are **a** the ribosomal A-site plazomicin binding site, and **b** the AAC(2′)-Ia acetylated-plazomicin binding site, colored as per Fig. 1. Depicted in both panels is a proposed extension to the 4′′ methyl group (light blue sphere), and a proposed hydroxyl substitution at the 2′ amine (red sphere) to plazomicin for next-generation aminoglycoside design.

While neither 2′ acetylation nor G1405 methylation are currently wide-spread mechanisms of aminoglycoside resistance, with continued usage of plazomicin, the incidence will inevitably rise. The three-dimensional structural data presented can provide helpful insights into the development of plazomicin derivatives with decreased susceptibility to resistance while maintaining antibiotic activity. Addressing modification of the 2′ amine group by AMEs is perhaps relatively straightforward through adding an extension at this location, analogous to how 6′ acetylation in plazomicin is prevented by the HE tail. The effectiveness of this strategy has been demonstrated in related 4,5-disubstituted aminoglycosides^32^. Alternatively, the 2′ amine group could be substituted by a hydroxyl, as is the case in amikacin and isepamicin, for example (Fig. 4). In theory, this substitution could be susceptible to 2′ phosphorylation or adenylation by AMEs, but enzymes with this activity have never yet been identified^29^. A concern with either of these approaches is that the overall positive charge of the antibiotic is reduced, which might negatively impact the affinity for the ribosome, as has been noted in the development of other next-generation aminoglycosides^32^. Our structural data reveals that despite the differences in van der Waals interactions between the ribosomal A-site and AAC(2′)-Ia (Fig. 2), there are very few synthetically feasible extensions to be made to the plazomicin structure that would provide another solution for preventing 2′ acetylation. One of the possibilities might be alterations at the 4′′ methyl location, which in the ribosome points away from helix 44, while in AAC(2′)-Ia an appropriate extension may create clashes with S116 (Fig. 4).

Addressing resistance conferred through G1405 methylation is perhaps even more challenging since this target alteration impacts binding of the plazomicin double-prime ring. There are aminoglycoside antibiotics that exploit the ribosomal A-site, which are unaffected by the presence of an m^7^G1405, i.e., 4,5-disubstituted aminoglycosides and unusual aminoglycosides such as the 4-monosubstituted apramycin. However, all of these lack the six-linked double-prime ring. Therefore, circumventing resistance by ribosomal methyltransferases, such as ArmA and RmtA will require a core structure that considerably departs from plazomicin.

## Conclusion

In summary, the structural data presented here reveals both the mechanism by which plazomicin exerts its antibiotic activity, as well as the structural basis for clinically observed resistance. The synthetic modifications made to the sisomicin scaffold afford plazomicin protection to nearly all of AMEs. However, this antibiotic is also not immune to resistance mechanisms. Our analysis reveals that further alteration to the scaffold may confer additional protection to drug modification. Unfortunately, avoiding resistance caused by target modification with the plazomicin scaffold appears unlikely. This highlights the importance of curtailing the spread of resistance while simultaneously expanding our armament of antibiotics.

## Methods

### Plazomicin synthesis

Synthesis of plazomicin was performed starting from commercially available sisomicin sulfate as recently reported^33^ in the modified version of the original report by Moser^15^.

### Ribosome purification

70S ribosomes were purified from HB8 *T. thermophilus* cells using the Selmer et al. purification protocol, with minor modifications to the final step. Here, zonal ultracentrifugation was replaced by three steps of 10–40% sucrose gradient preparation, ultracentrifugation, and fraction collection^19^.

### tRNA^fMet^ and tRNA^Phe^ expression

The tRNA^fMet2(MetY)^ and tRNA^PheV^ plasmids, encoded in pBS tRNA^fMet2^ and pBSTNAV2/tRNA^PheV^, respectively, were generously provided by Ramakrishnan Lab (MRC laboratory of molecular biology, UK) and Innis Lab (European Institute of Chemistry and Biology, France). The vectors were used to transform HMS174(DE3) competent cells. Cells were subsequently grown in 2YT medium at 37 °C for approximately 20 h. Cells were then harvested by centrifugation at 6000*g* for 15 min at 4 °C and resuspended in 1 mM TRIS-HCl, pH 7.5, and 10 mM magnesium acetate. The tRNA^bulk^ was extracted by organic RNA extraction method using a phenol solution saturated with 0.1 M citrate buffer, pH 4.3^34^. Amino acids bound to tRNA were removed by incubation in 1.5 M TRIS-HCl, pH 8.8, at 37 °C for 2 h.

### tRNA^fMet^ purification

The extracted tRNA^bulk^ was applied to a series of four HiTrap^™^ Q HP 5 mL columns (Cytiva) equilibrated in 20 mM TRIS-HCl, pH 7.5, 8 mM MgCl_2_, 200 mM NaCl, and 0.1 mM EDTA and eluted using a 20–35% gradient of equilibration buffer supplemented with 1 M NaCl^35^. tRNA^fMet^ fractions were identified using urea-PAGE and pooled. Pure tRNA^fMet^ was concentrated to approximately 100–150 μM and exchanged into a final storage buffer consisting of 10 mM ammonium acetate, pH 5.0, and 50 mM KCl using Amicon^®^ concentrators. tRNA^fMet^ was subsequently flash-frozen and stored at −80 °C until further use for complex formation.

### tRNA^Phe^ purification

tRNA^Phe^ was purified using the Junemann and Kayama methods^36,37^. Briefly, tRNA^bulk^ peak fractions from the anion-exchanged material were pooled and applied to the HiPrep^™^ Phenyl HP 16/10 column (Cytiva) equilibrated in 50 mM ammonium acetate, pH 5.3, 10 mM magnesium acetate, and 1.5 M ammonium sulfate and eluted using the same buffer in the absence of ammonium sulfate. Peak fractions containing tRNA^Phe^ were identified using urea–PAGE and pooled. The resulting material was then applied on a Symmetry300^™^ C4 (Waters) column equilibrated in 10 mM ammonium acetate, pH 5.5, 10 mM magnesium acetate, and 400 mM NaCl and eluted using equilibration buffer supplemented with 60% Methanol. tRNA^Phe^ was then precipitated using 3 M sodium acetate and 100% ice-cold ethanol in a 1:25 ratio. Pure tRNA^Phe^ was buffer exchanged, concentrated, and stored as described for tRNA^fMet^.

### mRNA Synthesis

The mRNA oligos with the sequence 5′-GGCAAGGAGGUAAAAAUGUUCUAA-3′ were chemically synthesized by Integrated DNA Technologies (Coralville, IA). The codons for tRNA^fMet^ and tRNA^Phe^ are underlined.

### Ribosome complex formation

Ribosome–mRNA–tRNA complexes were formed following the Polikanov et al. method^38,39^. Plazomicin was added to this complex with the final concentration of 125 μM during a 10-min equilibration step executed at 37 °C.

### Ribosome crystallization

Crystals of the 70S ribosome complex were grown at 19–21 °C using the sitting-drop vapor diffusion method. Drops contained a 1:1 ratio of the 70S ribosome complex and reservoir solution consisting of 100 mM TRIS-HCl, pH 7.6, 3–3.2% (w/v) PEG 20K, 7–12% (v/v) MPD, 100–200 mM arginine, and 0.5 mM β-mercaptoethanol. Crystals were sequentially transferred into a cryo-protecting solution consisting of 100 mM TRIS-HCl, pH 7.6, 3.2% PEG 20K, 10 mM magnesium acetate, 10 mM NH_4_Cl, 50 mM KCl, 6 mM BME, and 40% (v/v) MPD and flash-frozen in liquid nitrogen.

### Ribosome data collection, structure solution, and refinement

Diffraction data for optimized crystals of the 70S ribosome complex were collected at CMCF beamline 08ID-1 at the Canadian Light Source (100 K, 0.97857 Å). The dataset was then processed using the *xia2* pipeline^40^ [*DIALS*^41^]. The structure was determined using Fourier synthesis performed by *phenix.refine*^42^ using a previously solved 70S ribosome complex bound to paromomycin (PDB ID: 4V51) stripped of all non-protein and -RNA atoms. The structure was then refined by iterative cycles of reciprocal-space refinement with *phenix.refine* and real-space refinement and model building in *Coot*^43^. The ligand restraints for plazomicin were generated using *eLBOW*^44^. The missing bL36, uL10, and uL11 proteins from 4V51 were modeled using a second 70S ribosome complex (PDB ID: 4V5P). Final Ramachandran statistics are as follows: 67.3% favored and 13.0% outliers. The data collection and final refinement statistics of the model are listed in Table 1.

The final structure consists of the entire 70S ribosome in complex with its tRNA and mRNA ligands (except for the bL12 and bS1). The E-site is occupied with a noncognate tRNA, and the decoding region of the A-site is occupied by plazomicin. uL1, bL25, bL31, and uS2 were either poorly ordered or completely disordered.

### AAC(2′)-Ia cloning

The *aac(2*′*)*-*Ia* gene from *P. stuartii* was synthesized and subcloned into pET-15b expression vector between the *NdeI* and *BamHI* restriction sites with an N-terminal HIS-tag followed by a thrombin cleavage site and verified by DNA sequencing using the BioBasic Inc. gene synthesis service. The resulting vector was used to transform *E. coli* BL21(DE3) cells.

### AAC(2′)-Ia expression and purification

Protein expression was carried out using the Studier method for auto-induction, as previously described^25,45^. Cells were then harvested by centrifugation at 6000*g* for 15 min at 4 °C and resuspended in 40 mL of lysis buffer containing 50 mM TRIS-HCl, pH 8.0, 200 mM NaCl, 10 mM β-mercaptoethanol, 10% (v/v) glycerol and one EDTA-free protease inhibitor tablet (Roche). Cells were then lysed by sonication, and cell debris was subsequently removed by centrifugation at 50,000*g* for 30 min at 4 °C. The supernatant was further clarified by filtration through a 0.22 μm syringe-driven filter. The resulting material was applied on a 26 mm i.d. × 50 mm Ni-IDA-Sepharose^®^ column equilibrated in 50 mM TRIS-HCl, pH 8.0, 200 mM NaCl, 10 mM β-mercaptoethanol, 10% (v/v) glycerol and eluted stepwise with starting buffer supplemented with 150 mM imidazole. AAC(2′)-Ia containing fractions were identified by sodium dodecyl sulfate-polyacrylamide gel electrophoresis and pooled. In total, 50 µL of 1 unit µL^−1^ Thrombin was added to the pool and incubated overnight at 22 °C to remove the N-terminal HIS-tag. The pool was then applied on a HiTrap^™^ Benzamidine FF column (Cytiva) attached in series with the Ni-IDA-Sepharose^®^ column equilibrated in the aforementioned buffer to remove thrombin and the HIS-tag from the AAC(2′)-Ia sample. AAC(2′)-Ia fractions were desalted on HiPrep 26/10 Desalting column (Cytiva) equilibrated in 25 mM BIS–TRIS propane pH 7.5, 10 mM β-mercaptoethanol and 10% (v/v) glycerol. The desalted material was applied on DEAE Sepharose^®^ FF 26 mm i.d. × 140 mm column equilibrated in the identical buffer and eluted with 0–400 mM NaCl gradient over 16 column volumes. Peak fractions from the DEAE column were pooled, and buffer exchange was then performed on the same desalting column equilibrated in the final storage buffer consisting of 10 mM HEPES, pH 6.6, and 1 mM TRIS (2-carboxyethyl) phosphine hydrochloride (TCEP). AAC(2′)-Ia was then concentrated to 10 mg mL^−1^ and stored at 4 °C. Lastly, the enzymatic activity of the purified AAC(2′)-Ia was confirmed using a previously established assay^46^.

### AAC(2′)-Ia crystallization

Crystals of the AAC(2′)-Ia-acetylated plazomicin-CoA complex were grown at 4 °C using the sitting-drop vapor diffusion method. Drops contained a 1:1 ratio of 10 mg mL^−1^ of AAC(2′)-Ia in storage buffer supplemented with 10 mM acetyl-CoA and 10 mM plazomicin. Crystals of the AAC(2′)-Ia complex grew when reservoir solution consisted of 0.2 M LiCl and 40% (v/v) MPD.

### AAC(2′)-Ia data collection, structure solution, and refinement

Diffraction data for optimized crystals of the AAC(2′)-Ia-acetylated plazomicin–CoA complex were collected at CMCF beamline 08ID-1 at the Canadian Light Source (100 K, 0.97857 Å). The dataset was then processed using the *xia2* pipeline^40^ [*CCP4*^47^, *POINTLESS*^48^, and *XDS*^49^]. The structure was determined using Fourier synthesis performed by *phenix.refine*^42^ using a concurrently solved acetylated netilmicin–CoA complex stripped of all non-protein atoms. The structure was then refined by iterative cycles of reciprocal-space refinement with *phenix.refine* and real-space refinement and model building in *Coot*^43^. The ligand restraints for CoA and acetylated plazomicin were generated using *eLBOW*^44^. Final Ramachandran statistics are as follows: 98.2% favored, no outliers. The data collection and final refinement statistics of the model are listed in Table 1.

### Reporting summary

Further information on research design is available in the Nature Research Reporting Summary linked to this article.

## Supplementary information

Supplementary Information

Reporting Summary

## Data Availability

Structure of *T. thermophilus* ribosome in complex with Plazomicin (PDB ID: 7LH5). Structure of AAC(2′)-Ia in complex with CoA and Acetylated Plazomicin (PDB ID: 6VOU). Any remaining information can be obtained from the corresponding author upon reasonable request.
