# Supplementary material for: Structural basis for plazomicin antibiotic action and resistance
Source: Commun Biol. 2021 Jun 11;4:729. doi: 10.1038/s42003-021-02261-4 (PMC8195987; doi:10.1038/s42003-021-02261-4)
Supplement: Supplementary file 1 — Supplementary Information [file 42003_2021_2261_MOESM1_ESM.pdf]

Structural Basis for Plazomicin Antibiotic Action and Resistance

Tolou Golkar[a,b,1], Angelia V. Bassenden[a,b,1], Krishnagopal Maiti[c], Dev P. Arya[c], T. Martin Schmeing[a,b], Albert M. Berghuis[a,b,d,*]

[a]Department of Biochemistry, McGill University, McIntyre Medical Building, 3655 Promenade Sir William Osler, Montréal, Québec, Canada, H3G 1Y6

[b]Centre de Recherche en Biologie Structurale, McGill University, Bellini Life Science Complex, 3649 Promenade Sir William Osler, Montréal, Québec, Canada, H3G 0B1

[c]Department of Chemistry, Clemson University, Clemson, South Carolina, 29634, United States

[d]Department of Microbiology and Immunology, McGill University, Duff Medical Building, 3775 University Street, Montréal, Québec, Canada, H3A 2B4

[1]These authors contributed equally to this work

*Albert M. Berghuis, McIntyre Medical Sciences Building, 3655 promenade Sir-William-Osler, Room 905D, Montreal, Quebec H3G 1Y6

Email: albert.berghuis@mcgill.ca

Figure S1. Discovery maps of ribosome and AAC(2′)-Ia ligands.

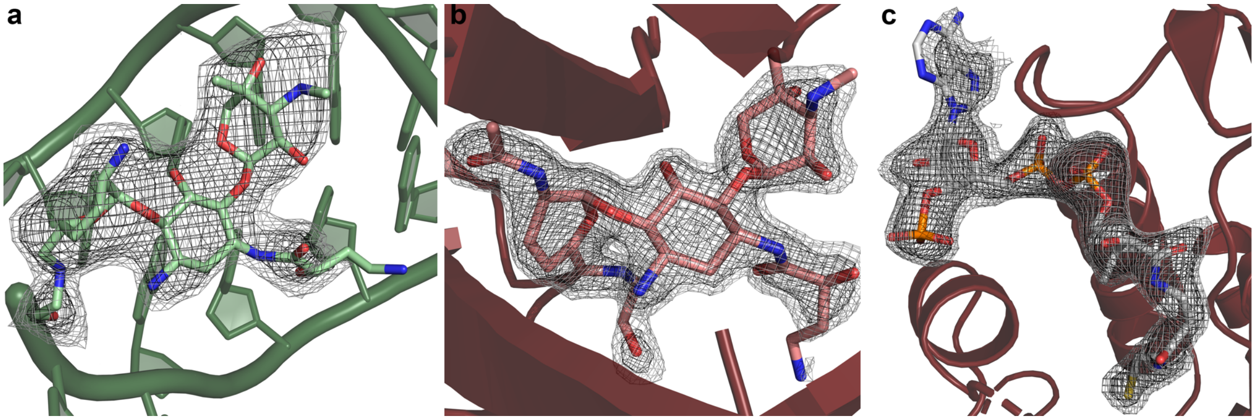

(a) View of the ribosomal A-site (dark green) bound to plazomicin (light green). (b) View of AAC(2′)-Ia (dark red) bound to acetylated plazomicin (salmon). (c) View of AAC(2′)-Ia bound to CoA (light grey). The F_o-F_c discovery maps are contoured at 3σ (dark grey) and 2σ (light grey), respectively, in all panels. Note, discovery maps are calculated in the early stages of refinement, prior to the inclusion of the modelled ligands. As these maps do not contain model bias with respect to ligands, they can be used the confirm the presence of these ligands in the structure.

Figure S2. Plazomicin bound to the 70S bacterial ribosome in complex with mRNA and tRNAs.

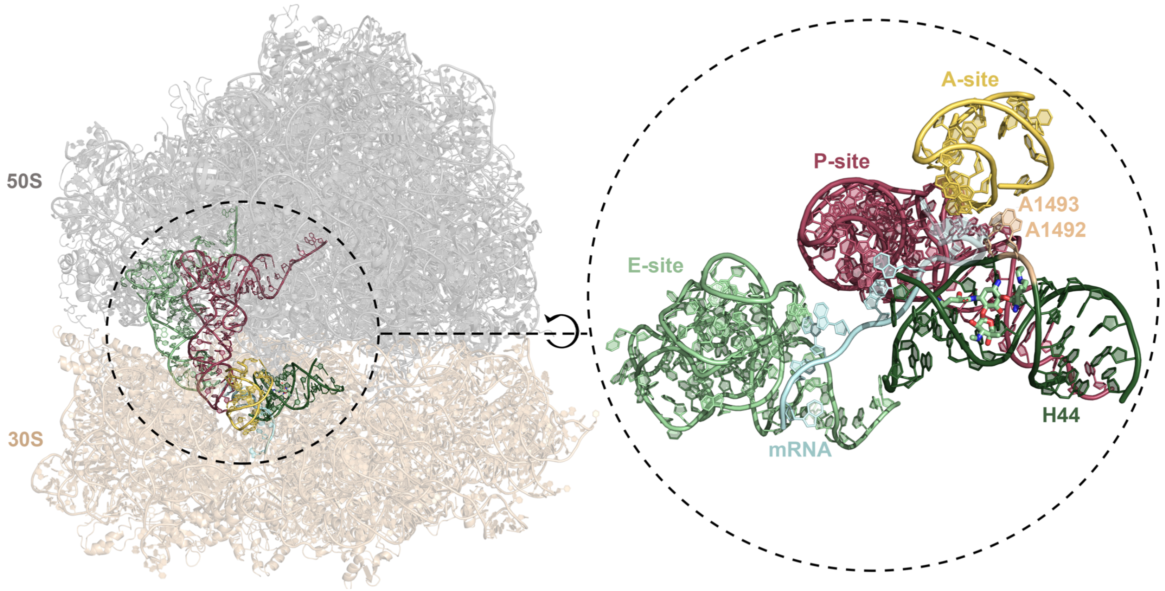

Panel insert shows details of the A-site (yellow), P-site (maroon), E-site (light green), Helix-44 (dark green), Helix-44, residue 1492 and 1493 (wheat) and mRNA (light blue), as well as the plazomicin binding site.

Figure S3. Structural comparison of apo and plazomicin-bound *T. thermophilus* ribosomal A-site.

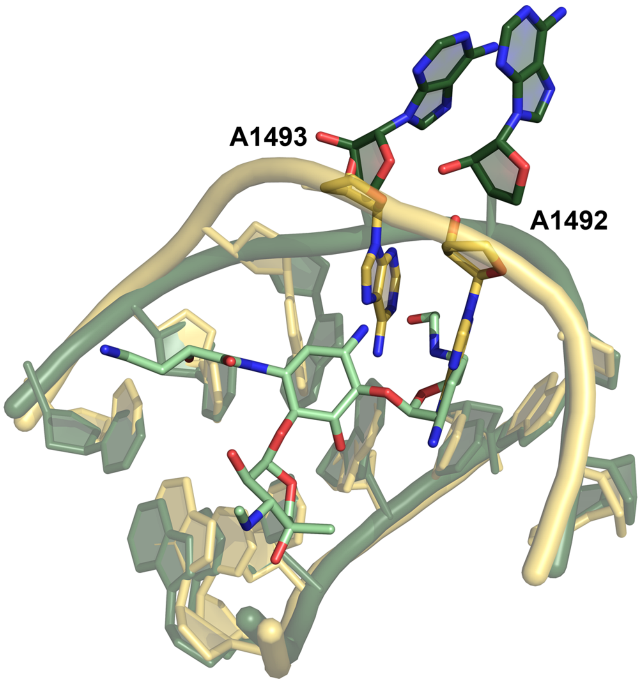

The ribosomal A-site apo structure (yellow, PDB ID: 1FKA) and the plazomicin-bound structure (dark green) are depicted, where residues A1492 and A1493 are shown in stick representation, highlighting their displacement upon plazomicin (light green) binding.

Figure S4. Alignment of aminoglycosides bound to the ribosomal A-site.

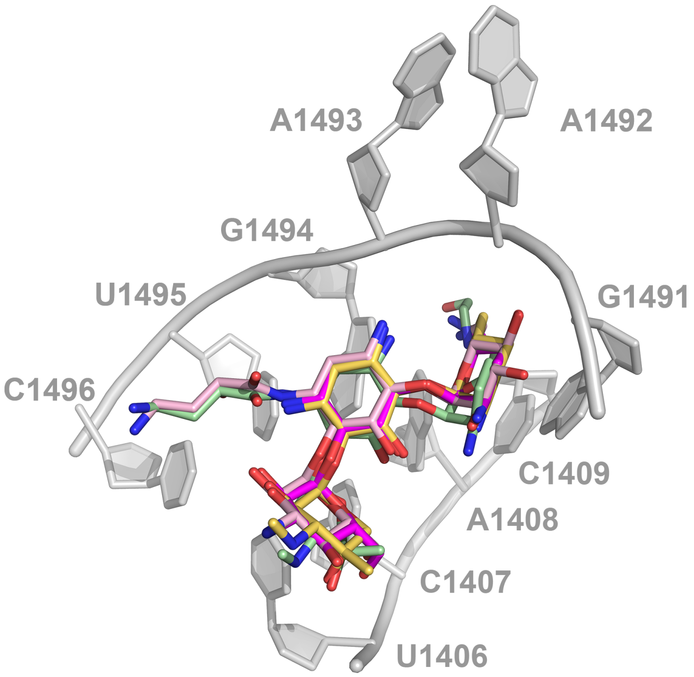

Depicted are amikacin (light pink, PDB ID: 4P20), gentamicin (yellow, PBD ID: 2ET3), plazomicin (light green), and tobramycin (magenta, PDB ID: 1LC4). The ribosomal A-site is shown in grey.

Figure S5. Model of plazomicin bound to helix 64 of the ribosome.

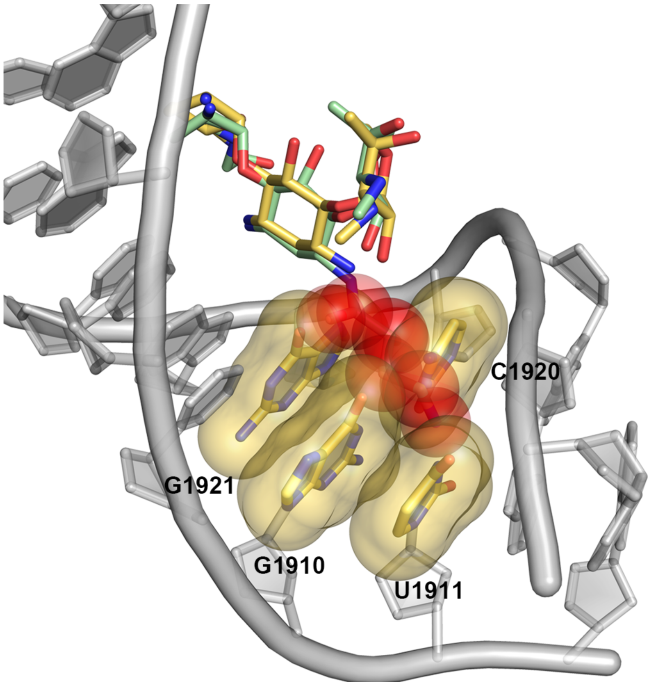

Model based on gentamicin-bound structure (PDB ID: 4V53). The ribosome structure is colored in grey, and bases predicted to clash with plazomicin are colored in yellow and depicted as surfaces. Gentamicin is shown in yellow, while the modelled plazomicin is shown in light green. The HABA tail of plazomicin is shown as red spheres.
